# Improved trends in cardiovascular complications among subjects with type 2 diabetes in Korea: a nationwide study (2006–2013)

**DOI:** 10.1186/s12933-016-0482-6

**Published:** 2017-01-06

**Authors:** Chang Hee Jung, Jin Ook Chung, Kyungdo Han, Seung-Hyun Ko, Kyung Soo Ko, Joong-Yeol Park, on Behalf of the Taskforce Team of Diabetes Fact Sheet of the Korean Diabetes Association

**Affiliations:** 1 Department of Internal Medicine, Asan Medical Center, University of Ulsan College of Medicine, 88 Olympic-ro 43-gil, Seoul, Songpa-gu 05505 South Korea; 2 Department of Internal Medicine, Chonnam National University Medical School, Kwangju, South Korea; 3 Department of Biostatics, The Catholic University of Korea, Seoul, South Korea; 4 Department of Internal Medicine, St. Vincent’s Hospital, College of Medicine, The Catholic University of Korea, Suwon, South Korea; 5 Depatment of Internal Medicine, Cardiovascular and Metabolic Center, Inje University Sanggye Paik Hospital, Inje University College of Medicine, 1342 Dongil-ro, Seoul, Nowon-gu 139-707 South Korea

**Keywords:** Type 2 diabetes, National, Cardiovascular disease, Complications, Trend

## Abstract

**Background:**

Representative data on the secular trends in cardiovascular disease (CVD) are limited in Asian populations with diabetes. We aimed to estimate the temporal trends in cardiovascular complications using Korean nationwide whole population-based claims data in subjects with and without diabetes.

**Methods:**

Type 2 diabetes was defined as a current medication history of anti-diabetic drugs and the presence of International Classification of Diseases (ICD)-10 codes (E11–E14) as diagnosis. We compared the 8-year rates of six cardiovascular complications [i.e., ischemic heart disease, acute myocardial infarction (AMI), ischemic stroke, hemorrhagic stroke, percutaneous coronary intervention (PCI), and coronary artery bypass graft (CABG)] in Korean adults aged 30 years and older using data from four consecutive nationwide databases (2006–2007, 2008–2009, 2010–2011, and 2012–2013) of Korean national health insurance service.

**Results:**

A total of 1,645,348, 1,971,559, 2,291,247, and 2,562,612 subjects with type 2 diabetes were found in the year of 2006–2007, 2008–2009, 2010–2011, and 2012–2013, respectively. Age and gender standardized rates of the six predefined cardiovascular complications decreased in Korean adults with type 2 diabetes during the study period. The greatest relative reductions were observed for hospitalization due to AMI (−37.28%), followed by hospitalizations due to ischemic stroke (−36.98%). In the overall population without type 2 diabetes, the greatest relative reductions were observed for hospitalization for hemorrhagic stroke (−29.47%), followed by hospitalization due to ischemic stroke (−28.92%). Relative decreases in all six predefined cardiovascular complications were generally more profound in adults with diabetes than in those without diabetes, which led to significant decrease in the relative risks of all six cardiovascular complications in subjects with diabetes over the past 8 years. However, people with diabetes still had a two- to sixfold higher risk of hospitalization for major CVD events and interventions than people without diabetes.

**Conclusions:**

Our findings suggest a significant reduction in the rate of people affected by CVD within the diabetic population. However, as the number of people with diabetes rises, the absolute burden of CVD will still be high in Korea.

**Electronic supplementary material:**

The online version of this article (doi:10.1186/s12933-016-0482-6) contains supplementary material, which is available to authorized users.

## Background

Diabetes is one of the most common metabolic disorders in the world and its prevalence in adults has been increasing in recent decades [1]. It has been estimated that the number of people in the United States with diagnosed diabetes will increase by 165% from 2000 to 2050, from 11 million to 29 million [2]. However, there are considerable variations in the burden of diabetes across regions, with developing countries disproportionately affected [1].

Diabetes is a well-known risk factor for cardiovascular disease (CVD) and is associated with a two- to fourfold increase in the risk of coronary artery disease development [3–5]. Fortunately, intensive glycemic control implemented in the early stage of diabetes as well as multifactorial risk management that includes better control of high blood pressure and dyslipidemia show beneficial effects on macrovascular complications and related mortality in subjects with diabetes [6, 7]. These studies were followed by a steady improvement in diabetes care and risk factor control [8–10]. Indeed, recent studies have reported decreases in cardiovascular complications in patients with diabetes, which have been attributed to better risk factor management [11–14]. For instance, Booth et al. [13] demonstrated a significant reduction in the rate of people affected by CVD within the diabetic population using provincial health claims data between 1992 and 2000. More recently, Gregg et al. [11], showed that the rates of diabetes-related complications declined substantially between 1990 and 2010, with the largest relative decline in acute myocardial infarction (AMI). However, most studies have been conducted in Western populations.

Representative data on the secular trends of diabetes-related cardiovascular complications are limited in Asian populations, particularly in Korea, where striking ethnic differences in CVD risk might exist [15], and diabetes is a major health problem with a rapidly increasing prevalence—from 1.5 to 9.9%—over the past 40 years [16]. The last decade has shown tremendous advances in the treatment of heart disease and CVD risk factors in Korea [17], as in Western countries [13, 18, 19].

Despite these improvements, it remains unclear whether cardiovascular complications have uniformly decreased among Korean populations with diabetes. Therefore, the aim of our present study was to estimate the temporal trends in major cardiovascular complications requiring hospital admission and cardiovascular interventions in Korean people with and without diabetes using nationwide whole population-based claims data. We also aimed to estimate the relative risk of these events in people with diabetes.

## Methods

### Data sources

A national health insurance system was initiated in 1963 in South Korea in response to the National Health Insurance Act, which mandated the participation of all citizens. Currently, the national health insurance service (NHIS) maintains and manages all databases of health service utilization in Korea. Briefly, the NHIS in Korea is a single-payer organization that is mandatory for all residents in Korea. Because it has adopted a fee-for-service model to pay health care providers who treat or examine Korean patients, NHIS obtains information on patient demographics, medical use/transaction information, insurers’ payment coverage, and patients’ deduction and claims databases (diagnosis, prescription, and/or consultation statements). Therefore, the NHIS database represents the entire Korean population and can be used as a population-based database. Further details of this database have been described previously [20, 21]. The National Health Insurance Sharing Service manages the NHIS database by operating three organizations within the “Big Data Steering Department” to maintain the quality of the database such as the “Information Analysis Division”, the “Data Convergence Division” and the “Information Analysis Division” [22].

In the present analysis, retrospective data of individuals older than 30 years of age were extracted from the Korean NHIS database between January 2006 and December 2013. The NHIS provides data without individual identifiers, in accordance with the Act on the Protection of Personal Information Maintained by Public Agencies. Thus, the database included an unidentifiable code representing each individual. Because this study was based on data from the NHIS, informed consent was not specifically obtained from the individuals. This study was approved by the Korean National Institute for Bioethics for Bioethics Policy (P01-201504-21-005).

### Definition of type 2 diabetes

Considering the characteristics of the NHIS database, an operational definition of diabetes was applied to the analysis. In this present study, individuals were defined as having diabetes if anti-diabetic drugs (insulins, sulfonylureas, metformin, meglitinides, thiazolidinediones, dipeptidyl peptidase-4 inhibitors, and α-glucosidase inhibitors) were prescribed with the presence of the International Classification of Diseases, 10th revision (ICD-10) codes E11 (non-insulin-dependent diabetes mellitus), E12 (malnutrition-related diabetes mellitus), E13 (other specified diabetes mellitus), or E14 (unspecified diabetes mellitus), as either principal diagnosis or additional diagnosis [20].

### Definition of cardiovascular complications

We identified incident cases of six diabetes-related cardiovascular complications: ischemic heart disease, AMI, ischemic stroke, hemorrhagic stroke, percutaneous coronary intervention (PCI), and coronary artery bypass graft (CABG). To identify cases of ischemic heart disease, AMI, ischemic stroke, and hemorrhagic stroke, we used the hospital discharge records based on ICD-10 codes as a principal diagnosis. The specific codes used were as follows: for ischemic heart disease, ICD-10 codes of I20 (angina pectoris), I21 (ST elevation and non-ST elevation myocardial infarction), I22 (subsequent ST elevation and non-ST elevation myocardial infarction), I23 (certain current complications following ST elevation and non-ST elevation myocardial infarction), I24 (other acute ischemic heart diseases), and I25 (chronic ischemic heart disease); for AMI, ICD-10 codes of I21, I22, and I23; for ischemic stroke, ICD-10 codes of I63 (cerebral infarction), I64 (stroke, not specified as hemorrhage or infarction), I693 (sequelae of cerebral infarction), I694 (sequelae of stroke, not specified as hemorrhage or infarction), and G45 (transient cerebral ischemic attacks and related syndromes); and for hemorrhagic stroke, ICD-10 codes of I60 (subarachnoid hemorrhage), I61 (intracerebral hemorrhage), I62 (other nontraumatic intracranial hemorrhage), I690 (sequelae of subarachnoid hemorrhage), I691 (sequelae of intracerebral hemorrhage), and I692 (sequelae of nontraumatic intracranial hemorrhage). We ascertained the incident cases of PCI and CABG by identifying the procedure codes of PCI (M6551-2, M6561-4, and M6571-2) and CABG (O1641-2, O6147, OA641-2, and OA647) [23]. We excluded any patients with a history of the six defined diabetes-related cardiovascular complications between 2002 and 2005.

To investigate possible explanations for the changing patterns of the six cardiovascular complications in subjects with or without type 2 diabetes, we analyzed the trends of hypertension and dyslipidemia, the two most representative risk factors for cardiovascular complications [24]. Individuals were defined as having hypertension if anti-hypertensive medications were prescribed with the presence of the ICD-10 codes I10 (essential hypertension), I11 (hypertensive heart disease), I12 (hypertensive renal disease), I13 (hypertensive heart and renal disease) or I15 (secondary hypertension). Similarly, individuals were defined as having dyslipidemia if lipid lowering agents were prescribed with the presence of the ICD-10 codes E78 (disorders or lipoprotein metabolism and other lipidemias).

### Statistical analysis

All rates are expressed as the number of events per 10,000 persons per year and the 95% confidence interval (CI), with age and gender standardized to Korean Census data for the year 2010 and the use of six age groups: 30–39 years, 40–49 years, 50–59 years, 60–69 years, 70–79 years and 80 years or more. Changes in cardiovascular complications between 2006 and 2013 were presented as percent change. Because the claims can be made in the month after the actual admission for CVD and the procedures for PCI and CABG, we calculated the number of events within a two years period (i.e., 2006 and 2007, 2008 and 2009, 2010 and 2011, and 2012 and 2013). We used the generalized linear model to test for linear trends of each cardiovascular complication over time. We estimated the relative risk (RR) and 95% CI of each cardiovascular complication in diabetic populations compared with those without diabetes using Poisson regression. Statistical analyses were performed using SAS version 9.2 (SAS Institute, Cary, NC). A *P* value <.05 was considered statistically significant.

## Results

### Diabetes rates

A total of 1,645,348, 1,971,559, 2,291,247, and 2,562,612 subjects with type 2 diabetes were found in the year of 2006–2007, 2008–2009, 2010–2011, and 2012–2013, respectively. The prevalence of type 2 diabetes increased from 5.6% in the year of 2006–2007 to 7.7% in the year of 2012–2013. The distribution of study participants from 2006 to 2013 by gender and age group (i.e., aged <65 years and ≥65 years) is indicated in Additional file 1: Table S1.

### Rates of cardiovascular complications according to the status of diabetes

**Fig. 1:**
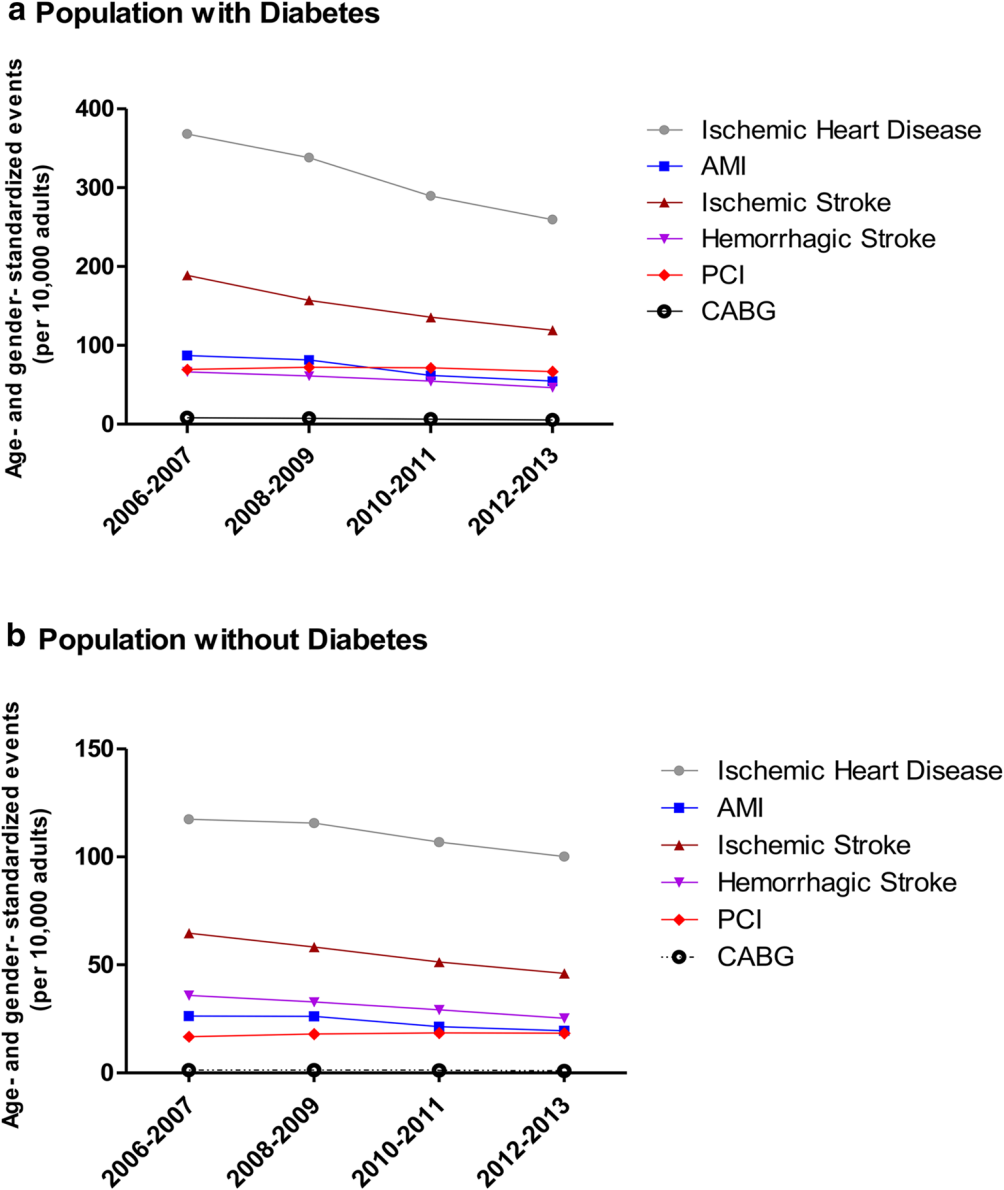
Age and gender standardized events (per 10,000 adults) of six cardiovascular complications in populations **a** with diabetes, and **b** without diabetes

As shown in Tables 1, 2, 3, 4, 5 and 6, the age and gender standardized rates of six predefined cardiovascular complications decreased in Korean adults with type 2 diabetes during the study period. In the overall population with type 2 diabetes, the greatest absolute reduction was in the number of hospitalizations due to ischemic heart disease (−108.45 fewer cases per 10,000 persons, Table 1; Fig. 1a), followed by hospitalizations due to ischemic stroke (−69.87 fewer cases per 10,000 persons, Table 3; Fig. 1a), AMI (−32.47 fewer cases per 10,000 persons, Table 2; Fig. 1a), hemorrhagic stroke (−20.01 fewer cases per 10,000 persons, Table 4; Fig. 1a), PCI (−2.59 fewer cases per 10,000 persons, Table 5; Fig. 1a), and CABG (−2.56 fewer cases per 10,000 persons, Table 6; Fig. 1a), respectively. When expressed in terms of the absolute number of cases (i.e., irrespective of changes in population size), the numbers of hospitalizations due to ischemic heart disease, AMI, ischemic stroke, hemorrhagic stroke, PCI, and CABG increased by 9879 cases, 876 cases, 847 cases, and 1623 cases, 9932 cases, and 194 cases respectively, from 2006 to 2013 (data not shown).

**Table 1:** Age and gender standardized rates of ischemic heart disease among Korean adults according to the presence or absence of diabetes (2006–2013)

Variables	Year				Change		P value
	2006–2007	2008–2009	2010–2011	2012–2013	Absolute change	Percent change	Linear trend
Ischemic heart disease							
Overall population							
No. of events/10,000 in diabetes	368.02	338.28	289.65	259.57	−108.45	−29.47	<.0001
95% CI	360.53–375.51	331.81–344.75	284.36–294.95	254.74–264.41			
No. of events/10,000 in non-diabetes	117.44	115.74	106.79	100.23	−17.21	−14.65	<.0001
95% CI	117.34–117.54	115.65–115.83	106.72–106.87	100.16–100.30			
RR (95% CI)	3.13 (3.10–3.17)	2.92 (2.89–2.95)	2.71 (2.68–2.74)	2.59 (2.56–2.62)			<.0001
Men							
No. of events/10,000 in diabetes	376.91	351.86	307.31	276.17	−100.74	−26.73	<.0001
95% CI	364.49–389.33	340.89–362.82	298.38–316.23	268.04–284.31			
No. of events/10,000 in non-diabetes	120.53	119.2	111.25	104.91	−15.62	−12.96	<.0001
95% CI	120.31–120.74	119.01–119.39	111.08–111.41	104.77–105.06			
RR (95% CI)	3.13 (3.09–3.16)	2.95 (2.92–2.98)	2.76 (2.73–2.79)	2.63 (2.61–2.66)			<.0001
Women							
No. of events/10,000 in diabetes	359.75	325.64	273.22	244.12	−115.63	−32.14	<.0001
95% CI	342.59–376.90	311.01–340.27	261.22–285.21	233.15–255.10			
No. of events/10,000 in non-diabetes	114.57	112.51	102.65	95.87	−18.7	−16.32	<.0001
95% CI	114.38–114.76	112.34–112.68	102.50–102.80	95.74–95.99			
RR (95% CI)	3.14 (3.11–3.17)	2.89 (2.86–2.93)	2.66 (2.63–2.69)	2.55 (2.52–2.57)			<.0001
Aged < 65 years							
No. of events/10,000 in diabetes	280.04	262.22	226.39	205.71	−74.33	−26.54	<.0001
95% CI	268.84–291.24	252.26–272.19	218.48–234.30	198.49–212.92			
No. of events/10,000 in non-diabetes	65.41	65.27	61.08	58.66	−6.75	−10.32	<.0001
95% CI	65.35–65.48	65.22–65.33	61.03–61.13	58.62–58.71			
RR (95% CI)	4.28 (4.23–4.33)	4.02 (3.97–4.06)	3.71 (3.67–3.75)	3.51 (3.47–3.55)			<.0001
Aged ≥ 65 years							
No. of events/10,000 in diabetes	820.52	732.68	617.92	534.07	−286.45	−34.91	<.0001
95% CI	755.38–885.67	690.16–775.2	590.45–645.4	515.73–552.41			
No. of events/10,000 in non-diabetes	404.83	402.36	372.02	340.52	−64.31	−15.89	<.0001
95% CI	401.01–408.65	398.89–405.82	369.09–374.96	338.15–342.89			
RR (95% CI)	2.03 (2.01–2.05)	1.82 (1.8–1.84)	1.66 (1.64–1.68)	1.57 (1.55–1.58)			<.0001

In the whole analysis, P values for interaction between diabetes status and years was less than .0001 by regression models

**Table 2:** Age and gender standardized rates of AMI among Korean adults according to the presence or absence of diabetes (2006–2013)

Variables	Year				Change		P value
	2006–2007	2008–2009	2010–2011	2012–2013	Absolute change	Percent change	Linear trend
AMI							
Overall population							
No. of events/10,000 in diabetes	87.09	81.36	61.67	54.62	−32.47	−37.28	<.0001
95% CI	85.27–88.91	79.82–82.89	60.64–62.69	53.71–55.54			
No. of events/10,000 in non-diabetes	26.31	26.15	21.43	19.59	−17.21	−25.50	<.0001
95% CI	26.29–26.33	26.13–26.17	21.42–21.45	19.57–19.60			
RR (95% CI)	3.31 (3.24–3.38)	3.11 (3.05–3.18)	2.88 (2.82–2.94)	2.79 (2.73–2.85)			<.0001
Men							
No. of events/10,000 in diabetes	100.97	92.90	73.55	66.12	−34.85	−34.52	<.0001
95% CI	97.37–104.58	89.84–95.95	71.34–75.77	64.16–68.08			
No. of events/10,000 in non-diabetes	30.34	29.51	24.13	22.27	−8.07	−26.60	<.0001
95% CI	30.29–30.39	29.46–29.56	24.09–24.16	22.24–22.30			
RR (95% CI)	3.33 (3.26–3.4)	3.15 (3.08–3.21)	3.05 (2.98–3.11)	2.97 (2.91–3.03)			<.0001
Women							
No. of events/10,000 in diabetes	74.17	70.61	50.60	43.92	−30.25	−40.78	<.0001
95% CI	70.52–77.81	67.52–73.69	48.69–52.51	42.21–45.62			
No. of events/10,000 in non-diabetes	22.55	23.01	18.92	17.09	−5.46	−24.21	<.0001
95% CI	22.52–22.59	22.98–23.05	18.89–18.95	17.07–17.11			
RR (95% CI)	3.29 (3.21–3.37)	3.07 (3.00–3.14)	2.67 (2.61–2.74)	2.57 (2.51–2.63)			<.0001
Aged < 65 years							
No. of events/10,000 in diabetes	66.35	63.53	47.05	42.57	−23.78	−35.84	<.0001
95% CI	63.62–69.08	61.09–65.98	45.57–48.52	41.20–43.93			
No. of events/10,000 in non-diabetes	14.12	14.25	11.63	10.70	−3.42	−24.22	<.0001
95% CI	14.11–14.13	14.24–14.27	11.62–11.64	10.69–10.71			
RR (95% CI)	4.70 (4.59–4.81)	4.46 (4.36–4.56)	4.05 (3.95–4.15)	3.98 (3.88–4.08)			<.0001
Aged ≥ 65 years							
No. of events/10,000 in diabetes	202.04	186.77	145.69	122.44	−79.60	−39.40	<.0001
95% CI	185.21–218.87	175.20–198.33	138.61–152.77	118.07–126.81			
No. of events/10,000 in non-diabetes	100.86	101.48	84.69	75.72	−25.14	−24.93	<.0001
95% CI	99.83–101.88	100.54–102.41	83.98–85.41	75.17–76.28			
RR (95% CI)	2.00 (1.96–2.05)	1.84 (1.8–1.88)	1.72 (1.68–1.76)	1.62 (1.58–1.65)			<.0001

In the whole analysis, P values for interaction between diabetes status and years was less than .0001 by regression models

**Table 3:** Age and gender standardized rates of ischemic stroke among Korean adults according to the presence or absence of diabetes (2006–2013)

Variables	Year				Change		P value
	2006–2007	2008–2009	2010–2011	2012–2013	Absolute change	Percent change	Linear trend
Ischemic stroke							
Overall population							
No. of events/10,000 in diabetes	188.94	157.29	135.46	119.07	−69.87	−36.98	<.0001
95% CI	186.41–191.46	155.28–159.29	133.75–137.18	117.51–120.63			
No. of events/10,000 in non-diabetes	64.72	58.25	51.29	46.00	−18.72	−28.92	<.0001
95% CI	64.66–64.78	58.21–58.30	51.25–51.33	45.96–46.03			
RR (95% CI)	2.92 (2.88–2.96)	2.70 (2.66–2.74)	2.64 (2.60–2.68)	2.59 (2.55–2.63)			<.0001
Men							
No. of events/10,000 in diabetes	188.65	159.79	141.53	125.13	−63.52	−33.67	<.0001
95% CI	184.30–193.00	156.42–163.16	138.67–144.39	122.68–127.58			
No. of events/10,000 in non-diabetes	63.64	57.35	51.63	46.93	−16.71	−26.26	<.0001
95% CI	63.52–63.75	57.25–57.44	51.55–51.70	46.86–46.99			
RR (95% CI)	2.96 (2.92–3.01)	2.79 (2.75–2.83)	2.74 (2.7–2.78)	2.67 (2.63–2.7)			<.0001
Women							
No. of events/10,000 in diabetes	189.2	154.96	129.82	113.43	−75.77	−40.05	<.0001
95% CI	183.56–194.84	150.39–159.52	125.90–133.73	109.74–117.12			
No. of events/10,000 in non-diabetes	65.73	59.1	50.98	45.13	−20.60	−31.34	<.0001
95% CI	65.62–65.85	59.01–59.19	50.91–51.05	45.07–45.19			
RR (95% CI)	2.88 (2.84–2.92)	2.62 (2.59–2.66)	2.55 (2.51–2.58)	2.51 (2.48–2.55)			<.0001
Aged < 65 years							
No. of events/10,000 in diabetes	104.76	91.84	82.45	75	−29.76	−28.41	<.0001
95% CI	101.91–107.61	89.24–94.43	80.20–84.71	72.91–77.09			
No. of events/10,000 in non-diabetes	22.98	21.07	19.66	18.12	−4.86	−21.15	<.0001
95% CI	22.96–23.00	21.05–21.09	19.64–19.67	18.10–18.13			
RR (95% CI)	4.56 (4.48–4.64)	4.36 (4.28–4.44)	4.19 (4.12–4.27)	4.14 (4.07–4.21)			<.0001
Aged ≥ 65 years							
No. of events/10,000 in diabetes	653.11	523.37	433.24	368.51	−284.6	−43.58	<.0001
95% CI	595.24–710.98	490.38–556.36	412.47–454.00	354.72–382.30			
No. of events/10,000 in non-diabetes	315.28	291.7	253.99	228.35	−86.93	−27.57	<.0001
95% CI	312.09–318.47	288.99–294.41	251.83–256.15	226.62–230.08			
RR (95% CI)	2.07 (2.05–2.1)	1.79 (1.77–1.82)	1.71 (1.68–1.73)	1.61 (1.59–1.63)			<.0001

In the whole analysis, P values for interaction between diabetes status and years was less than .0001 by regression models

**Table 4:** Age and gender standardized rates hemorrhagic stroke among Korean adults according to the presence or absence of diabetes (2006–2013)

Variables	Year				Change		P value
	2006–2007	2008–2009	2010–2011	2012–2013	Absolute change	Percent change	Linear trend
Hemorrhagic stroke							
Overall population							
No. of events/10,000 in diabetes	66.44	61.22	54.65	46.43	−20.01	−30.12	<.0001
95% CI	65.09–67.8	60– 62.45	53.57–55.72	45.5–47.36			
No. of events/10,000 in non-diabetes	35.94	32.85	29.23	25.35	−10.59	−29.47	<.0001
95% CI	35.91–35.97	32.83–32.88	29.21–29.25	25.33–25.37			
RR (95% CI)	1.85 (1.80–1.89)	1.86 (1.82–1.91)	1.87 (1.83–1.91)	1.83 (1.79–1.87)			<.0001
Men							
No. of events/10,000 in diabetes	69.60	64.00	58.65	47.92	−21.68	−31.15	<.0001
95% CI	67.48–71.73	62.04–65.96	56.96–60.33	46.57–49.27			
No. of events/10,000 in non-diabetes	37.65	34.6	31.14	26.83	−10.82	−28.74	<.0001
95% CI	37.59–37.72	34.55–34.66	31.10–31.19	26.8 –26.87			
RR (95% CI)	1.85 (1.8–1.89)	1.85 (1.81–1.89)	1.88 (1.84–1.93)	1.79 (1.75–1.83)			<.0001
Women							
No. of events/10,000 in diabetes	63.5	58.64	50.93	45.04	−18.46	−29.07	<.0001
95% CI	60.28–66.71	55.78–61.51	48.38–53.47	42.73–47.35			
No. of events/10,000 in non-diabetes	34.35	31.22	27.45	23.97	−10.38	−30.22	<.0001
95% CI	34.30–34.41	31.18–31.27	27.41–27.49	23.94–24.00			
RR (95% CI)	1.85 (1.80–1.90)	1.88 (1.83–1.93)	1.86 (1.81–1.90)	1.88 (1.83–1.93)			<.0001
Aged < 65 years							
No. of events/10,000 in diabetes	51.91	48.43	42.66	37.41	−14.5	−27.93	<.0001
95% CI	50.05–53.78	46.69–50.17	41.10–44.22	35.96–38.86			
No. of events/10,000 in non-diabetes	23.34	20.79	18.48	16.18	−7.16	−30.68	<.0001
95% CI	23.32–23.36	20.77–20.81	18.46–18.49	16.16–16.19			
RR (95% CI)	2.22 (2.17–2.28)	2.33 (2.27–2.39)	2.31 (2.25–2.37)	2.31 (2.25–2.37)			<.0001
Aged ≥ 65 years							
No. of events/10,000 in diabetes	145.8	132.03	122.85	101.88	−43.92	−30.12	<.0001
95% CI	133.07–158.54	123.83–140.22	116.69–129.00	98.04–105.72			
No. of events/10,000 in non-diabetes	106.26	103.24	94.47	82.17	−24.09	−22.67	<.0001
95% CI	105.23–107.29	102.33–104.15	93.7–95.24	81.58–82.77			
RR (95% CI)	1.37 (1.34–1.41)	1.28 (1.25–1.31)	1.3 (1.27–1.33)	1.24 (1.21–1.27)			<.0001

In the whole analysis, P values for interaction between diabetes status and years was less than .0001 by regression models

**Table 5:** Age and gender standardized rates of PCI among Korean adults according to the presence or absence of diabetes (2006–2013)

Variables	Year				Change		P value
	2006–2007	2008–2009	2010–2011	2012–2013	Absolute change	Percent change	Linear trend
PCI							
Overall population							
No. of events/10,000 in diabetes	69.52	72.12	71.58	66.93	−2.59	−3.73	<.0001
95% CI	68.56–70.48	71.26–72.98	70.72–72.45	66.16–67.70			
No. of events/10,000 in non-diabetes	16.86	18.00	18.59	18.43	1.57	9.31	<.0001
95% CI	16.84–16.87	17.98–18.01	18.58–18.6	18.42–18.44			
RR (95% CI)	4.12 (4.03–4.22)	4.01 (3.93–4.09)	3.85 (3.78–3.92)	3.63 (3.57–3.70)			<.0001
Men							
No. of events/10,000 in diabetes	91.56	94.84	95.28	91.72	.16	.17	.0343
95% CI	88.89–94.22	92.49–97.18	93.03–97.52	89.54–93.9			
No. of events/10,000 in non-diabetes	23.5	25.13	26.39	26.64	3.14	13.36	<.001
95% CI	23.46–23.55	25.09–25.17	26.35–26.42	26.60–26.68			
RR (95% CI)	3.9 (3.81–3.98)	3.77 (3.7–3.85)	3.61 (3.54–3.68)	3.44 (3.38–3.5)			<.001
Women							
No. of events/10,000 in diabetes	49.00	50.98	49.53	43.85	−5.15	−10.51	<.001
95% CI	47.73–50.27	49.80–52.15	48.25–50.80	42.89–44.82			
No. of events/10,000 in non-diabetes	10.67	11.36	11.33	10.79	.12	1.12	.784
95% CI	10.65–10.69	11.34–11.38	11.31–11.35	10.77–10.80			
RR (95% CI)	4.59 (4.46–4.73)	4.49 (4.37–4.61)	4.37 (4.26–4.49)	4.06 (3.96–4.17)			<.001
Aged < 65 years							
No. of events/10,000 in diabetes	52.51	53.43	53.2	50.13	−2.38	−4.53	<.001
95% CI	51.30–53.73	52.30–54.55	52.09–54.31	49.14–51.13			
No. of events/10,000 in non-diabetes	9.78	10.09	10.2	10.05	.27	2.76	.002
95% CI	9.77–9.79	10.08–10.10	10.19–10.21	10.04–10.06			
RR (95% CI)	5.37 (5.23–5.51)	5.3 (5.17–5.42)	5.22 (5.10–5.33)	4.99 (4.88–5.10)			<.001
Aged ≥ 65 years							
No. of events/10,000 in diabetes	130.66	146.82	148.89	138.75	8.09	6.19	.0019
95% CI	122.64–138.69	139.54–154.11	143.04–154.74	134.53–142.97			
No. of events/10,000 in non-diabetes	49.31	56.65	62.66	63.47	14.16	28.72	<.001
95% CI	48.91–49.71	56.21–57.08	62.20–63.12	63.05–63.88			
RR (95% CI)	2.65 (2.58–2.72)	2.59 (2.54–2.65)	2.38 (2.33–2.43)	2.19 (2.14–2.23)	−2.59		<.001

In the whole analysis, P values for interaction between diabetes status and years was less than .0001 by regression models

**Table 6:** Age and gender standardized rates of CABG among Korean adults according to the presence or absence of diabetes (2006–2013)

Variables	Year				Change		P value
	2006–2007	2008–2009	2010–2011	2012–2013	Absolute change	Percent change	Linear trend
CABG							
Overall population							
No. of events/10,000 in diabetes	8.19	7.46	6.46	5.63	−2.56	−31.25	<.0001
95% CI	8.10–8.28	7.40–7.53	6.38–6.54	5.56–5.70			
No. of events/10,000 in non-diabetes	1.33	1.29	1.13	.976	−.35	−26.67	<.0001
95% CI	1.33–1.332	1.291–1.293	1.131–1.133	.975–.976			
RR (95% CI)	6.15 (5.76–6.57)	5.78 (5.43–6.15)	5.71 (5.36–6.08)	5.77 (5.41–6.15)			<.0001
Men							
No. of events/10,000 in diabetes	11.40	10.28	8.94	8.01	−3.39	−29.74	<.0001
95% CI	11.16–11.64	10.09–10.48	8.75–9.12	7.86–8.17			
No. of events/10,000 in non-diabetes	2.02	1.94	1.67	1.462	−.56	−27.48	<.0001
95% CI	2.013–2.02	1.94–1.947	1.667–1.672	1.46–1.464			
RR (95% CI)	5.66 (5.3–6.04)	5.29 (4.97–5.63)	5.35 (5.02–5.7)	5.48 (5.14–5.84)			<.0001
Women							
No. of events/10,000 in diabetes	5.196	4.838	4.159	3.411	−1.79	−34.35	<.0001
95% CI	5.081–5.310	4.759–4.917	4.018–4.299	3.279–3.542			
No. of events/10,000 in non-diabetes	.692	.685	.632	.523	−.17	−24.42	<.0001
95% CI	.691–.694	.684–.686	.631–.633	.522–.523			
RR (95% CI)	7.51 (6.79–8.30)	7.06 (6.42–7.77)	6.58 (5.97–7.25)	6.52 (5.88–7.23)			<.0001
Aged < 65 years							
No. of events/10,000 in diabetes	5.807	5.157	4.567	3.91	−1.90	−32.67	<.0001
95% CI	5.709–5.906	5.079–5.235	4.467–4.667	3.825–3.995			
No. of events/10,000 in non-diabetes	.727	.686	.572	.505	−.22	−30.54	<.0001
95% CI	.726–.727	.685–.686	.572–.573	.505–.506			
RR (95% CI)	7.99 (7.37–8.66)	7.52 (6.96–8.12)	7.98 (7.37–8.65)	7.74 (7.13–8.41)			<.0001
Aged ≥ 65 years							
No. of events/10,000 in diabetes	15.749	14.518	12.417	10.875	−4.87	−30.95	<.0001
95% CI	14.895–16.603	14.01–15.026	12.055–12.779	10.641–11.109			
No. of events/10,000 in non-diabetes	3.645	3.77	3.552	3.101	−.54	−14.92	<.0001
95% CI	3.621–3.67	3.745–3.794	3.531–3.573	3.084–3.117			
RR (95% CI)	4.32 (4.01–4.66)	3.85 (3.59–4.13)	3.5 (3.26–3.75)	3.51 (3.26–3.77)			<.0001

In the whole analysis, P values for interaction between diabetes status and years was less than .0001 by regression models

In the overall population with type 2 diabetes, the greatest relative reductions observed were for hospitalization due to AMI (−37.28%, Table 2), followed by hospitalizations due to ischemic stroke (−36.98%, Table 3). When we performed subgroup analyses according to gender and age group (i.e., aged <65 years and ≥65 years) in Korean adults with type 2 diabetes, both genders showed similar patterns in the relative reductions, in which the greatest relative reduction were observed in following order; hospitalization due to AMI and ischemic stroke (−34.52 and −33.67% for men and −40.78 and −40.05% for women, as shown in Table 2 for AMI and Table 3 for ischemic stroke, respectively).

For adults with type 2 diabetes aged <65 years, the greatest relative reductions were observed for AMI (−35.84%, Table 2), followed by CABG (−32.67%, Table 6). For adults with type 2 diabetes aged ≥65 years, the hospitalization due to ischemic stroke showed the greatest relative reductions (−43.58%, Table 3), followed by AMI (−39.40%, Table 2).

In the overall population without type 2 diabetes, the greatest relative reductions observed were for hospitalization for hemorrhagic stroke (−29.47%, Table 4; Fig. 1b), followed by hospitalization due to ischemic stroke (−28.92%, Table 3; Fig. 1b). When we performed subgroup analyses according to gender and age groups (i.e., aged <65 years and ≥65 years) in Korean adults without type 2 diabetes, the greatest relative reductions among the six predefined cardiovascular complications were observed for hospitalizations due to hemorrhagic stroke for men (−28.74%, Table 4) and adults aged <65 years (−30.68%, Table 4), as well as ischemic stroke for women (−34.34%, Table 3) and adults aged ≥65 years (−27.57%, Table 3), respectively.

Regarding PCI, we observed some mixed patterns. Compared with other cardiovascular complications, all of which showed decreasing trends in relative reductions regardless of the status of diabetes as shown in Tables 1, 2, 3, 4 and 6, the relative statistically significant increase was observed in men regardless of the presence of diabetes (.17 and 13.36% for men with diabetes and without diabetes, respectively, Table 5) as well as in non-diabetic adults aged <65 years (2.76%) and all adults aged ≥65 years regardless of the presence of diabetes (6.19 and 28.72% for those with diabetes and without diabetes, respectively, Table 5).

### Trends in relative risks of cardiovascular complications in subjects with diabetes

**Fig. 2:**
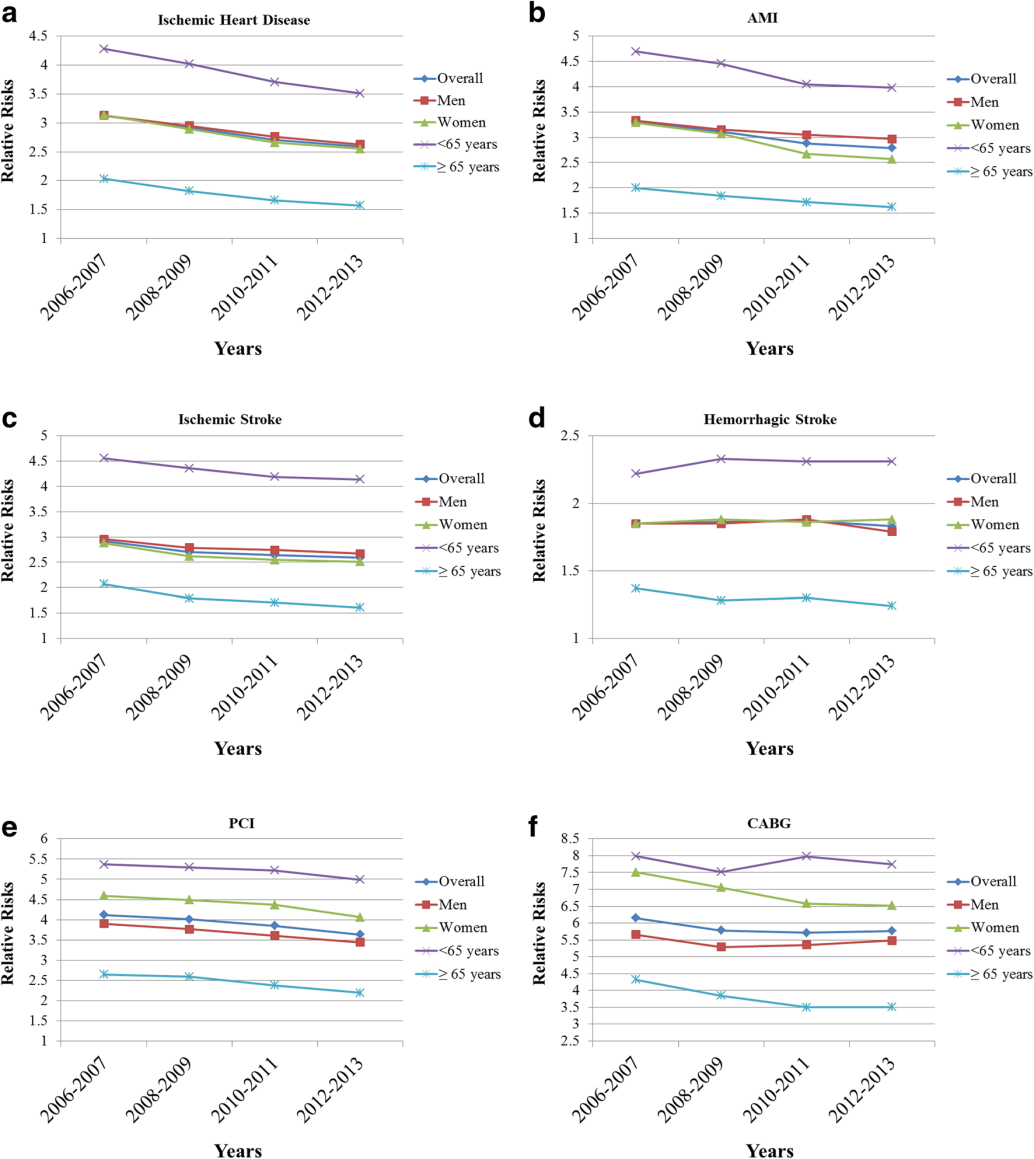
Relative risks of six cardiovascular complications in subjects with diabetes compared with subjects without diabetes. **a** Ischemic heart disease, **b** acute myocardial infarction (AMI), **c** ischemic stroke, **d** hemorrhagic stroke, **e** percutaneous coronary intervention (PCI), and **f** coronary artery bypass and graft (CABG)

The temporal trends in the relative risks of the six predefined cardiovascular complications in adults with type 2 diabetes are also shown in Tables 1, 2, 3, 4, 5 and 6, as well as in Fig. 2. For hospitalizations due to ischemic heart disease (Table 1; Fig. 2a), the relative reductions were more profound in adults with type 2 diabetes than in those without type 2 diabetes in overall population as well as in subgroup analyses according to gender and age groups. As a results, the relative risk of events associated with diabetes reduced from 3.13 (95% CI 3.10–3.17) during 2006–2007 to 2.59 (95% CI 2.56–2.62) during 2012–2013 (Table 1; Fig. 2a). Similar patterns in the relative risk of the hospitalization due to ischemic heart disease were observed across all subgroup analyses according to gender and age groups (Table 1; Fig. 2a).

The relative risks of other cardiovascular complications also showed similar patterns, in which the relative risks of hospitalization due to AMI decreased from 3.31 (95% CI 3.24–3.38) to 2.79 (95% CI 2.73–2.85, Table 2; Fig. 2b), hospitalization due to ischemic stroke decreased from 2.92 (95% CI 2.88–2.96) to 2.59 (95% CI 2.55–2.63, Table 3; Fig. 2c), hospitalization due to hemorrhagic stroke decreased from 1.85 (95% CI 1.80–1.89) to 1.83 (95% CI 1.79–1.87, Table 4; Fig. 2d), PCI decreased from 4.12 (95% CI 4.03–4.22) to 3.63 (95% CI 3.57–3.70, Table 5; Fig. 2e), and CABG decreased from 6.15 (95% CI 5.76–6.57) to 5.77 (95% CI 5.41–6.15, Table 6; Fig. 2f), respectively.

### Trends in the prevalence of hypertension and dyslipidemia according to the status of diabetes

**Fig. 3:**
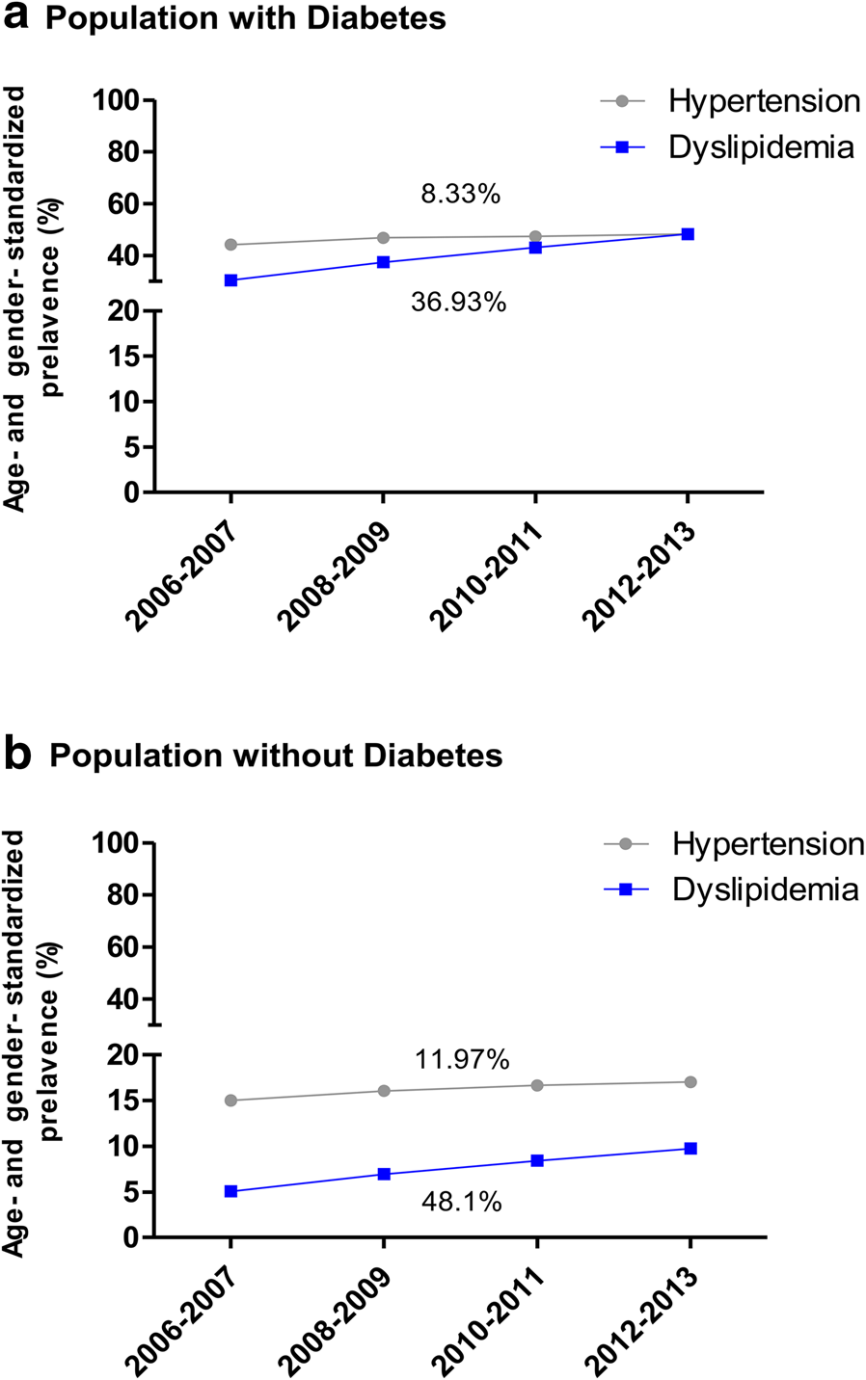
Age and gender standardized prevalence of hypertension and dyslipidemia in populations **a** with diabetes, and **b** without diabetes

We analyzed the age and gender standardized prevalence of treated hypertension and dyslipidemia in adults with and without type 2 diabetes during the study period. As shown in Fig. 3, the increasing trend in the prevalence of hypertension and dyslipidemia was more profound in subjects without diabetes compared with those with diabetes (11.97 vs. 8.33% for hypertension and 48.1 vs. 36.93% for dyslipidemia, respectively), although the absolute prevalence of both comorbidities was higher in subjects with diabetes compared with those without diabetes (Fig. 3).

## Discussion

Our current analysis of nationwide whole population-based claims data revealed a substantial relative reduction in the incidence of cardiovascular complications, with exception of PCI, in the Korean population between 2006 and 2013. Generally, in subjects with diabetes, hospitalization due to AMI (Table 2) and ischemic stroke (Table 3) accounted for the greatest relative reductions. Reductions in the rates were smallest for PCI, which actually increased among men and older adults (Table 5). Finally, the relative decreases in all six predefined cardiovascular complications were generally more profound in adults with diabetes than in those without diabetes (Tables 1, 2, 3, 4, 5 and 6), which led to significant decrease in the relative risks of all six cardiovascular complications in subjects with diabetes over the past 8 years (Tables 1, 2, 3, 4, 5 and 6; Fig. 2). However, people with diabetes continued to show a two- to sixfold higher risk of hospitalization for major CVD events and interventions than people without diabetes (Tables 1, 2, 3, 4, 5 and 6; Fig. 2). Our findings suggest a significant reduction in the rate of people affected by CVD within the diabetic population. However, as the number of people with diabetes rises (Additional file 1: Table S1), the absolute burden of CVD will remain high in Korea, highlighting the urgent need for comprehensive measures in preventing diabetes [25]. Our study is the first to show the recent admission rate for major CVD events and cardiovascular interventions in people with diabetes using a nationwide population claims database that covers nearly the entire population of Korea.

The importance of diabetes as a major cardiovascular risk factor has received considerable attention in the last decade [13]. Our findings of improved cardiovascular complications in the diabetic population support the findings of recent national survey- and registry-based studies in the US [11], as well as a nationwide study in England [26]. Numerous evidence-based interventions exist, ranging from glycemic and CVD risk factor control to early screening for diabetes complications [27]. These have been paralleled by population-wide improvements in glycemic control, CVD risk factors, and rates of several diabetes complications [28–30]. Indeed, favorable trends in the prevalence of hypertension, dyslipidemia, and cigarette smoking have been observed in Western countries [18, 19]. However, the evidence from Western populations cannot simply be extrapolated to Asian populations because there are ethnic differences in both the prevalence of cardiovascular risk factors and their association with diabetes [31].

In Korea, significant risk factor modifications, such as improved control of blood pressure and dyslipidemia as well as a decreased smoking rate, have also been observed in recent decades [17]. Although little is known about the secular trends in the control of hypertension among diabetic patients in Korea, recent data suggested better control of hypertension in the those population than in the non-diabetic population [32, 33]. Regarding dyslipidemia, the rates of awareness and treatment in diabetic patients were much higher than in non-diabetic individuals [34]. In line with these previous reports, we could identify that the increasing trend in the prevalence of hypertension and dyslipidemia under treatments was more profound in subjects without diabetes compared with those with diabetes (Fig. 3). Even though the increased uptake of community treatments substantially accounted for the reduction in CVD mortality [35], these improvements might have led to the overall significant reduction in cardiovascular complications in the population of Korean adults with diabetes compared with those without diabetes.

During the study period, we observed some mixed patterns in the number of PCI (Table 5). The relative statistically significant increase was observed in men regardless of the presence of diabetes as well as in non-diabetic adults aged <65 years and all adults aged ≥65 years regardless of the presence of diabetes (Table 5). This result is in accordance with those of other studies [26, 36], in which the PCI rate significantly increased due to marked advances in stent technology, such as the introduction of drug-eluting stents and adjunctive pharmacology [26, 36]. Although CABG offers more advantages in terms of survival and the need for repeat revascularization for patients with severe forms of coronary artery disease, particularly patients with diabetes [26], the aforementioned technological advances in PCI might have contributed to the shift from CABG to PCI in subjects with diabetes, as in individuals without diabetes.

When we compare the relative risk of major cardiovascular events and coronary interventions, the relative risks of hospitalization due to hemorrhagic stroke (i.e., less than 2.0 during the study period, Table 4; Fig. 2d) as well as the decline in the relative risks of hospitalization due to hemorrhagic stroke (i.e., 1.85 during 2006–2007 to 1.83 during 2012–2013, Table 4; Fig. 2d) were fairly small compared with other complications in individuals with diabetes. Diabetes is a risk factor mainly for ischemic stroke, though its association with hemorrhagic stroke remains controversial and depends on ethnicity [37]. In the Honolulu Heart Program, diabetes was not associated with an increased risk of hemorrhagic stroke in Japanese-American men, while in the Framingham study, there was a 4.5-fold excess risk of this type of stroke in white men with diabetes [38]. This relative lack of association of diabetes with hemorrhagic stroke at least in Asian populations might have resulted in the comparatively small relative risk, as well as the small decline of relative risk of hemorrhagic stroke in subjects with diabetes compared to those without diabetes in our analysis (Table 4).

There are several limitations to our study that need to be addressed. First, although the NHIS database represents the entire Korean population, one of the most critical drawbacks is the discrepancy between the diagnosis of individuals in real practice and that recorded in the claims database. However, the proportion of discrepancies in diagnosis is less prominent in claims data from in-patient hospitalization and procedure codes [20]. This was the reason why we defined cardiovascular complications based on hospital discharge and coronary intervention codes. Second, the operational definition of type 2 diabetes could be problematic because we defined diabetes based on the presence of both corresponding ICD-10 codes and claims for anti-diabetic drugs. Therefore, our results cannot be applied to subjects with undiagnosed diabetes and those with diabetes who are not taking any diabetic drugs. Third, we could not identify those with cardiovascular complications prior to 2002, because the Korean NHIS did not maintain and manage databases before 2002. Thus, there remains the possibility that incident events might be recurrent events in some subjects, if the events had occurred prior to 2002. Fourth, we could not obtain the mortality rate caused by predefined cardiovascular complications because the NHIS database does not present the cause of death [20]. Therefore, we could not ascertain that the reduced incidence of CVD events led to improved survival in subjects with and without diabetes. However, it is very important to investigate incidence for an accurate estimation of cardiovascular risk factors and the evaluation of population-based prevention program [39]. Finally, although our findings showed a substantial fall in CVD rates, we cannot attribute this change to one particular intervention over another. In a similar context, although we provided the subgroup analysis according to gender and age groups, we could not perform a detailed analysis according to comorbidities such as renal failure or obesity as well as socioeconomic status or region, all of which have been known to affect cardiovascular events and mortality in subjects with diabetes [40–44].

Despite the above limitations, one of the main strengths of our study is that we included all adults in the general population, allowing the study to reflect outcomes as they exist in the real world with minimal selection bias.

In conclusion, our findings indicate that the care and management of patients with diabetes have substantially improved in recent years in Korea and that efforts aimed at both primary and secondary prevention might have contributed to these favorable secular changes. However, as the number of people with diabetes rises, the absolute burden of CVD will continue to be high in Korea. Therefore, these findings emphasize the continuing need for aggressive risk reduction in people with diabetes.

## Additional file

**Additional file 1: Table S1.** Study participants (Korean NHIS beneficiaries aged ≥ 30 years) distributed by age and gender.

## Abbreviations

AMI: acute myocardial infarction
CABG: coronary artery bypass and graft
CI: confidence interval
CVD: cardiovascular disease
ICD-10: the International Classification of Diseases, 10th revision
NHIS: national health insurance service
PCI: percutaneous coronary intervention
