# Supplementary material for: Improved trends in cardiovascular complications among subjects with type 2 diabetes in Korea: a nationwide study (2006–2013)
Source: Cardiovasc Diabetol. 2017 Jan 6;16:1. doi: 10.1186/s12933-016-0482-6 (PMC5216535; doi:10.1186/s12933-016-0482-6)
Supplement: Supplementary file 1 — Additional file 1: Table S1. Study participants (Korean NHIS beneficiaries aged ≥ 30 years) distributed by age and gender. [file 12933_2016_482_MOESM1_ESM.docx]

**Supplementary Table S1**– Study participants (Korean NHIS beneficiaries aged ≥30 years) distributed by age and gender

| **Variables** | **Year** |  |  |  |
| --- | --- | --- | --- | --- |
|  | **2006–2007** | **2008–2009** | **2010–2011** | **2012–2013** |
| **Overall population** | 29174100 | 30717004 | 32077689 | 33268617 |
| No. of adults with diabetes (%) | 1645348 (5.6) | 1971559 (6.4) | 2291247 (7.1) | 2562612 (7.7) |
| No. of adults without diabetes (%) | 27528752 (94.4) | 28745445 (93.6) | 29786442 (92.9) | 30706005 (92.3) |
|  |  |  |  |  |
| **Men** | 14257661 | 15021165 | 15702207 | 16280584 |
| No. of adults with diabetes (%) | 864201 (6.1) | 1046145 (7.0) | 1226767 (7.8) | 1385902 (8.5) |
| No. of adults without diabetes (%) | 13393460 (93.9) | 13975020 (93.0) | 14475440 (92.2) | 14894682 (91.5 |
|  |  |  |  |  |
| **Women** | 14916439 | 15695839 | 16375482 | 16988033 |
| No. of adults with diabetes (%) | 781147 (5.2) | 925414 (5.9) | 1064480 (6.5) | 1176710 (6.9) |
| No. of adults without diabetes (%) | 14135292 (94.8) | 14770425 (94.1) | 15311002 (93.5) | 15811323 (93.1) |
|  |  |  |  |  |
| **Aged < 65 years** | 24582740 | 25673321 | 26647030 | 27275340 |
| No. of adults with diabetes (%) | 981041 (4.0) | 1147150 (4.5) | 1311573 (4.9) | 1423519 (5.2) |
| No. of adults without diabetes (%) | 23601699 (96.0) | 24526171 (95.5) | 25335457 (95.1) | 25851821 (94.8) |
|  |  |  |  |  |
| **Aged ≥ 65 years** | 4591360 | 5043683 | 5430659 | 5993277 |
| No. of adults with diabetes (%) | 664307 (14.5) | 824409 (16.3) | 979674 (18.0) | 1139093 (19.0) |
| No. of adults without diabetes (%) | 3927053 (85.5) | 4219274 (83.7) | 4450985 (82.0) | 4854184 (81.0) |
